# Supplementary material for: Are carbon emissions trading and green financial instruments synergistic? -Comprehensive quantitative research based on content analysis
Source: PLoS One. 2024 Mar 7;19(3):e0298601. doi: 10.1371/journal.pone.0298601 (PMC10919601; doi:10.1371/journal.pone.0298601)
Supplement: S1 Data — (ZIP) [file pone.0298601.s001.zip › Data/地级市Z_F工作报告.zip!/┴╔─■/▒╛╧¬╩╨/└┤╫╘╣1⁄2╓┌║┼í╛┬φ┐╦╩2╛▌═°í┐_2019.docx]

各位代表：

　　现在，我代表市人民政府向大会作报告，请予审议，并请市政协委员和各位列席会议的同志提出意见和建议。

　　一、2018年工作回顾

　　过去的一年，是本溪发展史上又一个不平凡、不容易、不简单的一年。一年来，在市委的坚强领导下，我们深入学习贯彻习近平新时代中国特色社会主义思想，全面贯彻党的十九大和十九届二中、三中全会精神，全面落实新发展理念和“四个着力”“三个推进”，坚持稳中求进工作总基调，贯彻实施“一带五基地”“五大区域发展战略”，坚定不移实施“工业强市、旅游兴市、生态立市”三大战略，以“重实干、强执行、抓落实”专项行动为主要抓手，抓项目促发展、抓稳定促和谐、抓党建促落实，较好地完成了各项目标任务，迈出了高质量发展的新步伐，实现了本届政府依法履职的良好开局。

　　从全年看，全市经济运行稳中有进、总体向好，主要有以下几个特点：

　　主要指标实现正增长。预计，地区生产总值813.2亿元，增长1.4%；一般公共预算收入62.6亿元，按可比口径增长12.7%；固定资产投资231.5亿元，增长10%；规模以上工业增加值254.7亿元，增长5%；社会消费品零售总额364.5亿元，增长2.5%。

　　投资结构发生积极变化。工业投资119亿元，占总投资比重达51.4%，增长71%，投资占比近五年来首次过半。尤其是制造业投资76.81亿元，占总投资比重34.4%，增长101%；高技术产业投资13.9亿元，占总投资比重6.2%，增长89.3%；民间投资134.94亿元，占总投资比重60.4%，增长27.5%。

　　一批重点项目工程竣工使用。投资20亿元的福耀玻璃两条生产线点火，投资15亿元的天士力东北现代中药示范工厂项目投产，投资7.2亿元的上药集团研发中试基地和人参精加工项目部分投产。投资10亿元的腾龙房车项目主体完工。投资8亿元的万达广场和投资2亿元的红星美凯龙开业运营。投资16.8亿元的“引观入本”输水工程，历时5年建设正式通水使用。辽中环线高速公路铁岭至本溪段、沈本线响山子至滨河南路段改扩建二期、集本线（本桓线）三架岭隧道及引线改建、田桓铁路和本宽线北大岭隧道等交通工程竣工通车。

　　人民生活水平稳步提高。坚持在发展中保障和改善民生，75%以上财政支出用于民生。确保了养老金按时足额发放。实现城镇新增就业2.3万人，城镇登记失业率为3.99%。基本退休金月人均增资128.6元。城镇常住居民人均可支配收入32861元，增长6%；农村常住居民人均可支配收入15485元，增长6.5%。

　　一年来，我们主要做了以下几方面工作：

　　（一）努力做强实体经济，发展新动能不断成长

　　坚定不移推动工业经济发展。全力服务本钢发展，市政府与本钢开展全面合作，20户地方采选企业向本钢供应铁精粉330万吨，启动建设恒达钢铁物流园，废钢铁加工配送基地挂牌运营，远通物资、钢联金属企业加快释放产能，辽宁吉和源100万吨废钢铁加工项目投产。高新区销售收入超亿元品种7个、超亿元企业30户，实现税收7.91亿元，其中纳税超千万元企业16户。全市规上工业企业实现主营业务收入1420亿元，增长17.8%；实现税收50亿元，增长24.6%。

　　加快促进现代服务业提档升级。大力创建全域旅游示范区，云山滑雪场、虎谷峡、大峡谷等项目建成运营。天著温泉、熙康云舍等重点项目加快建设。本溪水洞通过世界纪录认证，被誉为“世界最长可供连续乘船游览的地下暗河景区”。建设13处乡村民宿，建成48处旅游公厕。全年接待游客人数、旅游总收入保持10%以上增长。全市实现新增本外币贷款115.2亿元、社会融资总量124.5亿元。市商业银行成功更名为本溪银行，新增金融机构3家，新增新三板挂牌企业2家。

　　大力推进乡村振兴。以产业兴旺为引领，持续推进种植结构调整。落实北药产业发展规划，新增耕地中药材种植面积8630亩。全域种植黄精2100亩，发展刺五加18.1万亩。千亩软枣猕猴桃、裕祥明菌业木耳、云南农业大学院士工作站林下有机人参种植、同仁堂和天士力参茸等产业融合试点项目投产。新增林下药材和山野菜基地4.45万亩、干坚果经济林基地2.55万亩。新组建林业新型经营主体35个。3058名贫困人口、17个贫困村实现脱贫。

　　加快发展民营企业。制定出台了支持民营经济高质量发展的19条政策举措。落实国家减税降费政策减收3.9亿元，全年实现“个转企”431户、“小升规”21户。全市新增市场主体16810户，增长17.5%。

　　（二）加大改革开放力度，创新发展活力明显增强

　　机构改革顺利推进。市政府部门由42个减少到29个；全市参改的事业单位由1261家优化整合为123家，总体规模压缩90%以上。

　　不断深化国资国企改革。全市34户全民所有制企业全部完成公司制改制，完成4户企业混合所有制改革，完成僵尸企业处置年度任务和纳入省考核的 “三供一业”分离移交工作任务，有序推进厂办大集体改革。

　　持续优化营商环境。深入推进“放管服”改革，公布“最多跑一次”政务服务事项2011项，实现与省政务服务平台数据交换“一库管理、数据同源”731项。高新区“3550”集中审批在全市推广，“证照分离”改革在全市推开。落实“七个不允许”要求，严格执行企业评议部门单位服务制度，对不满意率超过5%的12个部门进行了约谈，曝光典型案例21起，处理责任人160人次。

　　全力以赴招商引资上项目。围绕钢铁、生物医药、新能源等十大重点领域全国50强企业，大力开展“走出去、请进来”，共签约项目114个，总投资391.8亿元，其中67个项目实现当年签约当年开工。建立领导干部包扶重点项目制度，推动了400个投资500万元以上项目加快实施。向上争取资金50亿元，增长10%。确定了各县区飞地经济园区，明确了高新区和桥北经济开发区两个市级飞地经济园区载体，出台了相关政策。积极融入沈阳经济区建设，深化与泰州市对口合作。

　　加大创新发展力度。全市科技型中小企业达到127家，实现科技成果转化71项。高新区获批国家打造科技支撑型特色载体，华润三药完成999小儿感冒颗粒划转并正式投产，上海绿谷治疗阿尔茨海默症新药完成三期临床并已向国家申请上市。

　　（三）加强生态环境建设，城乡面貌进一步改善

　　更加重视生态环境保护。拆改取缔10吨及以下燃煤锅炉332台，国电投热电联产项目投入运营，东部城区1200万平方米用户实现集中清洁供暖，空气优良天数比例达到90.7%，PM10和PM2.5平均浓度可达到国家二级标准。完成本溪县、桓仁县、南芬区污水处理厂提标改造。全面建立河长制。完成市级水源地专项整治，集中式饮用水源地水质达标率100%。完成彩屯河黑臭水体整治全线5.2公里管线铺设，南沙河污水截流工程开工建设。完成营造林12万亩。农用地土壤污染调查结果通过国家和省审核认定。坚决推进中央生态环保督察反馈意见整改落实，“回头看”中转办的群众信访投诉问题已办结99.3%。

　　不断提升城市整体形象。有效规范房地产市场秩序，完成未验收入住房屋确权76个项目、3.89万户，解决“回迁难”2210户。坚定实施主城区环境综合整治，开展城市管理年活动，坚决拆除违建、乱建，改造老旧小区101个，全面实施“三供一业”、早期棚改小区维修改造工程，推行自助式物业管理，小区面貌焕然一新。实施姚溪路等10条城市道路、114条街巷路、2座危桥改建工程。高标准改造站前游园，建设城乡公厕68座。积极推进绿化、亮化、美化工程，新装、维修路灯3600余盏，大力实施交通秩序整治，清理占道经营，彻底治理唐家路二手车市场。

　　建设生态宜居乡村。东营坊乡和向阳乡被评为省级特色乡镇。大力整治农村人居环境，完成“四好农村路”建设203.8公里、“一事一议”村内道路路面工程354.9公里。

　　（四）提升公共服务水平，不断增强人民群众的获得感幸福感安全感

　　切实加强社会保障。全市最低工资标准市区调整为1420元、两县调整为1300元。将低保和特困人员救助供养标准，城市提高6%、农村提高10%。调整城镇居民基本医保政府补助标准，每人每年提高30元。完成棚户区改造4724户。

　　推动社会事业全面进步。药都中心幼儿园被评为全国家园共育示范园。全市所有县区义务教育均衡发展均通过国检。本溪市机电工程学校通过国家级中等职业教育改革发展示范校验收。有序开展分级诊疗，全市建立了384个家庭医生签约服务团队，签约39.4万人。基层医疗服务机构标准化建设达标率50%以上。本溪湖工业遗产群文物保护工程全面启动。创建30个基层文化中心示范点。新市殡仪馆、市失能老人养护中心建成运营。

　　全力维护社会和谐稳定。实施平安本溪“民生警务”，构建网格化巡逻防控机制，深入推进扫黑除恶专项斗争，社会治安环境持续好转。加大信访矛盾减存控增力度，化解信访积案860件、化解率69.6%。妥善处置龙新矿业思山岭铁矿“6.5”炸药爆炸事故。有效开展了安全生产、公共安全、防汛抗旱、森林防火、非洲猪瘟防控等工作，保持了社会和谐稳定。积极支持国防和军队现代化建设，国防动员、人防和民兵预备役工作不断加强，双拥共建取得新成绩。

　　2018年，我们按照省政府的部署要求，开展了“重实干、强执行、抓落实”专项行动。围绕173项重点指标任务，与“三抓三促”有效衔接，攻下了一批项目、解决了一批问题、办成了一批大事，促进了干部作风持续好转。加强政府自身建设，认真履行全面从严治党和党风廉政建设主体责任，自觉接受市人大及其常委会的法律监督、工作监督和市政协的民主监督，497件市级建议提案全部办结，问题解决率72.4%。

　　回首过去的一年，尽管我们面临的形势异常复杂、面临的困难超乎想象、面临的风险空前严峻、面临的压力多重叠加，但凭着对党和人民满腔忠诚和担当，我们硬是开创了本溪发展的新局面。奋斗充满艰辛，成绩来之不易、难能可贵。取得这些成绩，得益于市委的坚强领导，得益于市人大、市政协的监督支持，得益于全市人民的戮力同心。在此，我代表市人民政府，向全市人民，向人大代表、政协委员、各民主党派、工商联和人民团体，向驻溪人民解放军指战员、武警部队官兵、公安干警，向中省直单位以及所有关心支持本溪发展的社会各界人士，一并表示崇高的敬意和衷心的感谢！

　　在看到成绩的同时，我们也要清醒认识到存在的问题和挑战，主要表现在：一是经济总量小，地区生产总值、一般公共预算收入绝对值等指标在全省排10位左右；经济结构失衡，国有经济比重大，民营经济不发达。二是产业结构单一偏重，生物医药、钢铁深加工产业完成产值占比分别仅为2.6%、3%，尚未真正形成新支撑，旅游产业发展还不充分。三是大项目少、好项目少，特别是缺少引领带动性强的工业大项目，新兴产业投资对经济发展拉动不足，且当前项目库储备亿元以上项目不到120个、总投资不到800亿元，面临着重大项目建设青黄不接的困境。四是历史遗留问题多，厂办大集体正常生产经营企业少，多数企业内欠问题严重，“僵尸企业”存量资产少，债务多，职工安置难，国资国企改革包袱沉重。五是营商环境建设任重道远，不同程度地存在政府承诺多兑现少、承诺快兑现慢、新官不理旧账的问题，严重影响了政府形象和发展环境。六是部分干部思想僵化、作风不实的问题比较突出，还存在不敢担当、不愿作为、精神懈怠的现象，存在多干多错、少干少错、不干不错的心态，存在“等靠要”的思想。对这些问题要认真加以克服和解决。

　　二、2019年工作安排

　　今年是新中国成立70周年，是全面建成小康社会关键之年，也是推进本溪振兴发展的攻坚之年，做好各项工作至关重要。当前，我国发展正处于并将长期处于重要战略机遇期，经济长期向好态势不会改变；辽宁正处于动能再造期、基础再建期、优势再创期，发展势头强劲；我们本溪经过几年来的扎实发展，新动能正不断集聚、新基础正不断夯实、新优势正不断增强。只有解放思想、更新观念，跳出本溪看本溪，才能抓住机遇、用足政策，闯出一条全面振兴、全方位振兴的新路；只有坚持深化供给侧结构性改革，在“巩固、增强、提升、畅通”上下功夫，才能不断推动本溪经济高质量发展；只有坚定信心、团结奋进，才能一步一步地向前走，把本溪自己的事情办好。

　　2019年工作总体要求：坚持以习近平新时代中国特色社会主义思想为指导，全面贯彻党的十九大和十九届二中、三中全会精神，深入贯彻落实习近平总书记在辽宁考察时和在深入推进东北振兴座谈会上重要讲话精神，全面落实中央和省委、市委经济工作会议精神，统筹推进“五位一体”总体布局，协调推进“四个全面”战略布局，认真落实“五个坚持”要求，补齐拉长“四个短板”，全力抓好“六项重点工作”，认真贯彻“一带五基地”和“五大区域发展战略”部署，以“三都”建设为突破，聚焦争先晋位目标，坚定不移实施“工业强市、文旅兴市、生态立市”发展战略，推进“抓党建促振兴、抓项目促发展、抓稳定促和谐”，继续打好三大攻坚战，统筹推进稳增长、促改革、调结构、惠民生、防风险工作，以新气象新担当新作为，保持经济持续健康发展和社会大局稳定，为全面建成小康社会收官打下决定性基础，以优异成绩庆祝中华人民共和国成立70周年。

　　2019年主要预期目标：地区生产总值增长与全省保持同步，一般公共预算收入增长8%左右，固定资产投资增长10%，规模以上工业增加值增长8%，社会消费品零售总额增长7%，城乡居民收入增长和经济增长基本同步。

　　今年要重点做好以下八个方面工作：

　　（一）不断壮大工业经济。工业始终是本溪经济发展的决定性力量，我们必须坚定不移、信心百倍地实施工业强市战略。

　　全力服务本钢提质增效。加快释放2000万吨优质钢材产能。重点推进60万吨高端优质线材生产线项目、本钢1700热轧完善改造项目、北营公司炼钢一区产能置换工程、特钢电炉升级改造工程、热镀锌高强钢及镀铝硅生产线工程、一冷轧改造工程和焦炉脱硫脱硝污染治理等项目开工建设，1号转炉节能环保改造等项目竣工投产，新建四冷轧工程等项目开展前期工作。全力支持服务本钢集团混改。

　　做深做细钢铁精深加工产业。推动大台沟铁矿探矿井建设、龙新矿业思山岭铁矿恢复建设、本溪东方三家子矿业铁精粉项目投产达效,地方采选企业向本钢全年供应铁精粉力争达到500万吨。重点推动本溪银龙年产10万吨预应力棒线材生产线、本溪金桥焊材年产18万吨高端焊条焊丝生产线及德科斯米尔、华岳精工等企业释放产能，辽宁民盛智能橡塑机械研发及产业化等项目竣工，加快恒达钢铁物流园建设，废钢铁加工配送基地力争实现产量150万吨，产能达到400万吨。全市钢铁深加工产业实现工业产值增长10%以上。

　　推进生物医药产业发展。坚定不移地推进高新区建设。推动恒康、药联、恒宝参药、祥云药业等重点企业加快释放产能，上海绿谷治疗阿尔茨海默症新药上市，好护士健康科技保健食品、国药中金龙参、上药人参精深加工等一批产业项目投产达效。推进上药集团研发中试基地、成大疫苗等续建项目加快建设，鼎持生物等项目开工建设。

　　提升智能制造水平。建设高强钢冷轧生产线国家智能制造示范等项目，力争10个以上新产品开发、关键技术研发、产品质量技术攻关项目列入省计划，认定市级以上企业技术中心3至5家。

　　加快民营企业发展。开展“服务企业年”活动，全面落实支持民营经济高质量发展19条政策，降低企业用地、用电、用工成本，电力市场交易电量占比达45%以上，金融机构对民营企业贷款连续三年每年新增100亿元，建立民营企业贷款担保基金，力争实现“个转企”400户、“小升规”40户、“规升巨”5户。构建亲清政商关系，大力弘扬企业家精神，保障企业家合法的人身和财产权益，保障企业合法经营，让民营企业安心放心创业发展。

　　（二）加快推动旅游等服务业发展。旅游是本溪的看家优势，我们必须持之以恒实施文旅兴市战略，大力建设全域旅游示范区。

　　强化旅游规划统筹。完善旅游规划体系，编制实施本溪市全域旅游规划和两县国际旅游度假区规划，着力打造全域旅游发展空间布局。与华侨城旅投集团开展战略合作，开发优质旅游资源。推行重点国有旅游景区混合所有制改革，组建本溪市旅游集团，推动全市旅游资源整合上市。

　　突出旅游特色发展。本溪的绿水青山和冰天雪地就是金山银山。做好“冰雪+温泉”文章，推动云山滑雪场二期、关东滑雪场一期、天著温泉、观山湖温泉等一批项目建成运营，策划推出回龙湖冬捕、冰上龙舟、雪乡民宿等冬季旅游项目。促进文旅融合发展，深入挖掘本溪地域文化，开发更具文化内涵的旅游商品，整合红色旅游、工业旅游资源，充分利用辽宁东北抗日义勇军纪念馆、辽宁东北抗联史实陈列馆、文物遗迹、世界文化遗产、非物质文化遗产等开展文化体验旅游，加快实施本溪湖工业遗产群一号高炉等保护修缮项目。丰富枫叶旅游内涵，推动枫林谷三期、大冰沟开发等项目加快建设。发展康养旅游业态，推进熙康云舍等项目建设。开发农业休闲观光旅游，打造田园综合体和田园艺术景观，培育精品乡村民宿10处以上。

　　健全旅游服务体系。精心打造旅游环线，加快干线公路与景区公路连接线及相邻景区之间公路建设。布局建设市县两级游客集散服务中心。建设枫都庄园等有档次的特色酒店。实施智慧旅游工程，建设旅游电子商务、大数据、应急指挥系统。城乡新建、改造标准化旅游公厕36座。

　　加强旅游宣传营销。实施品牌营销战略，加大运用新媒体宣传力度，塑造特色鲜明的“中国枫叶之都”“中国温泉之城”旅游目的地品牌形象，巩固优质客源市场，深度开发新的客源市场，全年旅游总收入保持12%左右增长。

　　大力发展商贸物流服务业。推动商贸企业线上线下融合发展。大力培育物流配送、电商等新业态，推进本溪商贸冷链物流二期、富虹商业综合体等项目建设。推进电商乡镇村建设，强化电商人才培养，培训800人次。

　　（三）全力抓好项目建设。本溪抓发展的重中之重就是抓项目，我们必须举全市之力谋划、引进、建设项目，争取有更多的大项目好项目落地，不断集聚发展后劲。

　　深入推进双招双引。积极开展“项目引进年”活动，紧密对接十大重点领域全国50强企业，实施精准招商，主动“走出去、请进来”，争取在钢铁精深加工、精密铸件、汽车零部件、生物医药等领域，引进重点项目120个以上。积极参与“一带一路”建设，深化与江苏泰州对口合作，全方位融入沈阳经济区，推进开放合作向纵深发展。实施“百千万人才计划”“专业技术人才知识更新工程”“高技能人才振兴计划”，积极引进高端人才、创新创业人才。

　　加快重点项目建设。推进天士力、腾龙房车、福耀玻璃第三条生产线等一批在建项目竣工投产运营，鑫暾生物质发电、康恩贝和辽宁参康源北药合作项目、金桥焊丝等一批签约项目开工建设，早期棚改、南芬城市供水等一批民生项目建成使用。

　　争取政策资金支持。抢抓国家新一轮振兴东北老工业基地政策机遇，包装储备一批项目，积极争取国家和省政策资金支持，全年向上争取资金增长10%以上。加快推进溪田铁路电气化提速改造、本桓（宽）高速公路、大雅河和太子河抽水蓄能电站、氢能产业园等项目前期工作。

　　大力发展飞地经济。结合发展基础、资源禀赋和特色优势，以飞地经济为载体，落实飞地经济园区政策，推动县域经济发展，力争两县建成飞地经济园区，各乡镇都要有飞地经济项目落地，不断培育壮大县域特色产业集群，推动县域产业转型升级。

　　（四）持续深化改革创新。本溪的发展就在于改革创新这关键一招，我们必须千方百计让本溪的发展更有动力、更加充满活力。

　　继续优化提升营商环境。进一步简政放权，深入实施“多证合一”“证照分离”改革，全面深化“互联网+政务服务”，全面推行马上办、网上办、就近办、一次办，提升审批服务规范化、智能化、便捷化水平。一般性经营企业开办时间压缩至3个工作日内，力争建设项目审批时限压减至90个工作日，不动产登记5天办结，高频事项实现“最多跑一次”，政务服务事项网上可办率不低于70%。向本溪桥北和本溪湖两个经济开发区下放市级审批权力。在规上企业中实行“项目管家”制度。

　　推进国资国企改革。加快推动国有企业混合所有制改革。完善国有企业法人治理结构，深化“三项制度”改革。完成僵尸企业处置任务。稳妥推进厂办大集体改革。

　　提高科技创新能力。围绕生物医药、钢铁精深加工重点产业布局，优化和完善重点实验室、产业技术创新平台，获批省级以上科研平台5家。构建产学研深度融合的技术创新体系,加强对中小企业创新支持,新增高新技术企业6家。促进形成市、县区科技成果转化体系，力争实现成果转化70项，有效专利拥有量达到410件以上。

　　（五）大力实施乡村振兴战略。做好“三农”工作、促进乡村振兴是本溪协调发展的重要内容，我们必须因地制宜、发挥优势、大力推进。

　　推动特色农业发展。持续推进山野菜、食用菌、优质米、冰葡萄等“一村一品”特色优势产品发展。促进中草药种植规模化、标准化，以黄精为主的中药材种植面积增长5%。林下药材基地新增3万亩，干坚果经济林基地新增2万亩。建设生态田园、生态药园，推进“三品一标”认证工作。市级农业龙头企业稳定在120家左右，农产品加工集聚区主营业务收入增长7%以上，加快形成农业特色化、规模化、品牌化、多样化发展格局。

　　深化农业农村改革。推进资源变资产、资金变股金、农民变股东改革。稳步推进和完善农村土地“三权分置”制度，推进家庭农场建设，确权登记颁证率达到90%以上。继续做好农村集体产权制度改革工作，推进组建村集体经济合作社。全面完成新一轮全国集体林业综合改革试验区建设任务，实现林地标准化、规模化、集约化经营，推动林下经济健康发展。

　　加快生态宜居乡村建设。加大农村人居环境整治力度，开展农村“六边”垃圾和“三堆”清理整治。统筹规划乡村供水、排水、道路、照明、能源供应、公厕等配套设施建设，高标准建设国家级特色小镇、省级特色乡镇。推进“四好农村路”建设，完成农村公路建设工程106公里。做好农村危房改造工作。

　　（六）不断提升城市建设管理水平。建设和谐宜居、富有活力、独具特色的美丽本溪，是我们大家共同的心愿，我们必须下大气力提升城市形象，把本溪建设得更加美好。

　　强化城市总体规划引领。开展城市总体规划论证报批，有效指导城市建设。推行“多规合一”，进一步完善城乡规划体系，启动城市交通等专项规划编制工作。严格规划审批，强化规划监管，切实提高城乡规划的科学性、权威性、严肃性。

　　推进城市基础设施建设。积极推进滨河北路、竖井路等道路维修改造，开工建设新溪湖大桥，推进太子河生态环境整治和防洪治理，完成威宁大街地下综合管廊和南沙河污水截流工程，维修峪明路二号桥和振兴桥等2座危桥，建设峪明路高峪过街天桥，完善城市生活垃圾和污泥无害化处理设施。

　　实施城市精细智慧管理。继续开展城市管理年活动，深化全市老旧小区综合整治，全面高质量完成早期棚改小区维修改造任务，积极推进本钢等国有企业家属住宅“三供一业”维修改造。因地制宜推动建立物业管理长效机制。实施“城市双修”，打造一批示范道路、示范社区、示范公园和示范亮化单位。深入推进城市数字化、精细化、网格化、智慧化管理，充分发挥数字化城市管理平台作用，建立快速反应机制，不断提高城市基础设施完好率和维修及时率。切实加强房地产市场管理，严格审批、有效监管，力争完成剩余未验收入住房屋确权和超期回迁工作任务，杜绝再发生此类问题。

　　持续改善生态环境。坚持“突破水、巩固气、治理土”，大力推进污染防治。继续加大水源地保护力度，全面做好桓仁水库、观音阁水库水源保护，完成两个水库保护区调整，推进集中式饮用水水源规范化管理。全面推行河长制，完成彩屯河黑臭水体整治和北沙河综合治理。启动城市建成区20吨及以下燃煤锅炉取缔工作，加强城市扬尘治理，落实六个百分之百要求，加大秸秆焚烧管控，环境空气质量达标率达到80%以上。开展营林造林，森林覆盖率保持在76%以上。有效管控农用地和城市建设用地土壤环境风险。继续加强农村人居环境整治，推进本溪县国家生态文明先行示范区建设。落实省中央环保督察整改年部署，以中央生态环保督察“回头看”为契机，狠抓反馈问题整改落实，让本溪的天更蓝、山更绿、水更清。

　　（七）持续增进民生福祉。政府工作的出发点和落脚点就是让人民过上好日子，我们必须始终坚持以人民为中心的发展思想，做到一切依靠人民、一切为了人民。

　　切实加强社会保障。抓好稳定就业,促进高校毕业生等重点群体就业，严格落实退役军人安置政策，加大力度促进退役军人就业创业，城镇新增就业2万人，城镇登记失业率控制在4.5%以内。抓好社会保障,提高城乡低保、特困供养标准不低于省定水平。落实新建住宅小区养老服务设施配建要求。推动民间资本参与养老机构建设。

　　促进社会事业发展。深化教育改革，整合优化教育资源，推进县域内城乡义务教育一体化发展，推动开展中小学课后服务。加快校企融合发展，完成2个职业教育实训基地项目建设。推进文化事业发展，完成市博物馆《山魂水魄》陈列改造项目，办好群众文化节。持续深化公立医院综合改革和医药卫生体制改革，健全现代医院管理制度和分级诊疗制度，不断提升医疗服务质量。实施两县农村及全市城镇困难家庭适龄妇女“两癌”免费检查。开展全民健身活动，加强基层公共体育设施建设，做好申办2024年全国冬运会分赛场工作。大力支持国防和军队建设，推进“双拥”工作创新发展。

　　打好精准脱贫攻坚战。大力开展脱贫“回头看”，继续实施“五个一批”工程，巩固脱贫成果，建立完善脱贫攻坚长效机制，确保实现“两不愁三保障”目标。

　　积极防范重大风险。采取有效措施，加强养老保险基金征缴，加大财政预算保障力度，确保养老金按时足额发放；加强科学管理，稳妥处置政府债务存量，严格防控政府债务风险；树立过紧日子的思想，坚持“先生活、后生产”，严格压缩一般性支出，有效防范财政支出风险。加强对地方中小银行、互联网金融等薄弱环节风险管控，防止发生系统性、区域性金融风险。

　　保持社会和谐稳定。不断创新社会治理方式，持续推进平安本溪“民生警务”，坚持打防并举、综合施治，严打各类违法犯罪，深化扫黑除恶专项斗争，健全网格化巡逻防控机制，从严公共安全管理。加大信访矛盾减存控增力度，加强社会矛盾排查，妥善化解影响社会稳定的突出问题。继续深入贯彻落实“七五”普法依法治市规划，开展“法律七进”“一村屯一社区一法律顾问”等活动。坚决落实安全生产党政领导责任、部门和属地监管责任、企业主体责任，继续推进安全生产网格化建设, 强化安全隐患排查整治，坚决遏制重特大安全事故发生。强化食品药品监管，切实抓好防汛抗旱、森林防火、“大棚房”整治等工作，营造更加平安和谐的社会环境。

　　（八）努力建设人民满意政府。坚持把“重实干、强执行、抓落实”专项行动作为主要抓手，不断加强政府自身建设，勇担发展重任，主动创新作为，建设人民满意政府。

　　强化政治建设。始终坚持政府工作讲政治、业务工作重党建，牢固树立“四个意识”、坚定“四个自信”、坚决做到“两个维护”，坚决在思想上政治上行动上同以习近平同志为核心的党中央保持高度一致，确保省委、省政府、市委的决策部署和工作要求落到实处、见到实效。加强政府系统党支部规范化建设。

　　坚守为民宗旨。加快补齐民生领域短板，下决心继续解决一批群众关心关注的切身利益问题，下决心继续解决一批企业反映的突出问题和历史遗留问题，努力满足人民群众对美好生活的向往和追求。

　　严格依法行政。严格执行重大事项集体决策制度，自觉接受市人大及其常委会的法律监督、工作监督和市政协的民主监督，全力办好人大代表议案建议、政协提案。政府要带头讲诚信，新官必须理旧账，作出的承诺必须坚决兑现，推动形成诚实守信的社会环境。

　　坚持真抓实干。聚焦争先晋位，与强者为伍、向先进看齐，解放思想、提振信心，奋勇争先、奋力赶超，大力开展“重强抓”专项行动，落实“三抓三促”要求，看准的事、认准的理，坚决要一抓到底、抓出成效。

　　保持清正廉洁。严格履行全面从严治党政治责任，持之以恒落实中央八项规定精神和市委实施细则，深化廉政风险防控，完善部门权责清单，加强审计监督，强化社会监督，守住党纪国法的“高压线”，做到秉公用权、廉洁用权，坚决维护本溪风清气正的良好政治生态。

　　各位代表，实干赢得未来，奋斗成就梦想。让我们高举习近平新时代中国特色社会主义思想伟大旗帜，在市委的坚强领导下，一起奋进，共同拼搏，全面完成今年各项目标任务，为让本溪人民生活得更加幸福而努力奋斗！
